# Targeting the Ras–Ral Signaling Axis in Type 2 Diabetes Mellitus: A Dual-Modulation Approach to Correcting Insulin Resistance and β-Cell Dysfunction

**DOI:** 10.3390/ph19040648

**Published:** 2026-04-21

**Authors:** Narayanan Thulasi, Kannan Harithpriya, Kumar Ganesan, Kunka Mohanram Ramkumar

**Affiliations:** 1 Department of Biotechnology, School of Bioengineering, SRM Institute of Science and Technology, Kattankulathur, Chennai 603203, Tamil Nadu, India; nm9366@srmist.edu.in (N.T.); khp.2201@gmail.com (K.H.); 2 School of Chinese Medicine, Li Ka Shing Faculty of Medicine, The University of Hong Kong, Hong Kong SAR, China; kumarg@hku.hk

**Keywords:** Ras GTPases, Ral GTPases, type 2 diabetes mellitus, insulin resistance, β-cell dysfunction, molecular therapeutics

## Abstract

Type 2 diabetes mellitus (T2DM) is driven by insulin resistance and β-cell dysfunction. While Ras GTPases are known for oncogenic signaling, emerging evidence implicates the Ras–Ral axis as a critical regulator of glucose homeostasis. This review synthesizes the distinct roles of Ras and Ral in metabolism. Ras hyperactivation promotes insulin resistance and inflammation via MAPK/PI3K pathways, whereas RalA supports GLUT4 translocation and insulin granule exocytosis. We propose a dual-pathway hypothesis: T2DM pathophysiology involves an imbalance characterized by excessive Ras signaling and insufficient Ral-mediated metabolic actions. Consequently, we explore the therapeutic potential of rebalancing this axis through combinatorial strategies, that selectively inhibit pathogenic Ras while enhancing protective Ral activity. We critically evaluate current Ras-targeted agents (e.g., farnesyltransferase inhibitors, allele-specific inhibitors) and discuss the emerging frontier of Ral-specific enhancers. Finally, we outline key translational challenges and future directions for validating this axis as a target for precision medicine in T2DM.

## 1. Introduction

Diabetes mellitus (DM) is a chronic metabolic disorder characterized by persistent hyperglycemia. Current global estimates indicate that nearly 500 million individuals are affected, with projections suggesting this number may rise to 800 million by 2045 [1]. The etiology and progression of diabetes involve complex metabolic imbalances stemming from both genetic predisposition and environmental factors. The disease primarily manifests in two forms: Type 1 DM (T1DM), resulting from the autoimmune-mediated destruction of pancreatic β-cells and consequent insulin deficiency, and Type 2 DM (T2DM), which is characterized by insulin resistance in peripheral tissues and progressive β-cell dysfunction. While the incidence of T1DM continues to increase globally at an annual rate of 2–5%, T2DM remains the most prevalent form in adults and is strongly associated with obesity, suboptimal dietary patterns, and sedentary lifestyles [2,3].

Insulin, the body’s principal anabolic hormone, orchestrates critical physiological processes, including glucose uptake, cellular growth, and survival, while concurrently inhibiting apoptosis [4]. Its biological effects are mediated through the insulin signaling cascade, which plays a central role in regulating systemic energy metabolism. A defining feature of diabetes pathophysiology is the impairment of insulin production due to β-cell dysfunction, leading to diminished cellular glucose uptake and sustained hyperglycemia [5]. Upon stimulation by insulin or insulin-like growth factors (IGFs), two major downstream pathways are activated: the phosphatidylinositol 3-kinase (PI3K)/AKT pathway, responsible for metabolic regulation, and the Ras/mitogen-activated protein kinase (MAPK) pathway, which governs mitogenic responses and cell proliferation [4].

The Ras family of small GTPases serves as crucial molecular switches, transducing extracellular growth signals to intracellular effectors and thereby regulating fundamental cellular processes such as proliferation, differentiation, and survival. Among the downstream effectors of Ras, the Ral GTPases (RalA and RalB) play a pivotal role in cellular functions, including vesicle trafficking, actin cytoskeleton dynamics, and cell proliferation [6]. Emerging evidence extends the significance of the Ras–Ral axis beyond its well-established role in oncogenic signaling, highlighting its involvement in metabolic regulation, where it influences glucose homeostasis and lipid metabolism [7]. Aberrant activation of this pathway has been implicated in the progression of various pathologies, including non-small cell lung cancer [8]. Given its dual role in cellular proliferation and metabolic regulation, a deeper understanding of the Ras–Ral signaling network may reveal novel therapeutic targets. The objective of this review is to comprehensively explore the role of the Ras–Ral axis in disease pathogenesis, with a particular emphasis on its emerging significance in the pathophysiology of DM and the potential for developing targeted interventions to manage metabolic dysfunction.

## 2. Ras-Ral: A Small GTPase Protein

### 2.1. Ras Protein

The Ras family comprises small GTPase proteins with a molecular weight of approximately 21–25 kDa, which function as pivotal regulators of diverse cellular signaling pathways governing proliferation, differentiation, and receptor activation [9]. Ras proteins act as binary molecular switches, cycling between an inactive guanosine diphosphate (GDP)-bound state and an active guanosine triphosphate (GTP)-bound state. This cycle is tightly controlled by regulatory proteins. Upon stimulation by upstream signals, guanine nucleotide exchange factors (GEFs)—notably Son of Sevenless homologs 1 and 2 (SOS1 and SOS2) catalyze the exchange of GDP for GTP, thereby activating Ras [10].

Activated Ras subsequently engages a network of downstream effectors, most prominently the PI3K/AKT and MAPK/extracellular signal-regulated kinase (ERK) pathways, which orchestrate essential cellular processes including growth, survival, and metabolism [11]. Dysregulation of Ras activity, frequently resulting from impaired GTP hydrolysis due to mutations, constitutes a fundamental driver in the pathogenesis of various conditions, including cancer and metabolic disorders [11].

The canonical Ras protein family includes three major isoforms—K-Ras, H-Ras, and N-Ras, which share significant structural homology and conserved GTPase function but exhibit distinct tissue distributions and biological roles [12]. Among these, K-Ras is the most frequently mutated isoform, with common activating mutations occurring at codons 12, 13, and 61. These mutations are strongly associated with oncogenesis and have also been implicated in metabolic dysregulation. In the context of diabetes, mutant K-Ras has been shown to disrupt canonical insulin signaling, thereby contributing to the development of insulin resistance and the pathogenesis of T2DM [13]. Although less commonly mutated, H-Ras has been implicated in diabetic pathophysiology through its activation of the Ras/Raf/MAPK pathway, which can impair insulin sensitivity [14]. N-Ras, predominantly expressed in hematopoietic cells, is commonly associated with hematological malignancies and melanoma; however, emerging evidence also suggests a potential role in metabolic dysfunction, indicating that its dysregulation may influence T2DM susceptibility [15].

### 2.2. Ral Protein

Ral (Ras-like) GTPases constitute a distinct subgroup within the Ras superfamily, sharing approximately 40–51% sequence similarity with Ras proteins and possessing a homologous G-domain structure. A defining structural feature of Ral is an N-terminal extension of 11 amino acids, which contributes to its unique regulatory functions [16]. Analogous to Ras, Ral proteins function as molecular switches, cycling between an inactive GDP-bound state and an active GTP-bound state through nucleotide-dependent conformational changes. Among the Ral isoforms, RalA and RalB are the most extensively characterized due to their high structural similarity to Ras, with divergence primarily localized to their C-terminal domains. Despite their close homology, the C-terminal regions of RalA and RalB contain distinct phosphorylation motifs that influence subcellular localization and confer isoform-specific biological activities [17]. Both isoforms interact with a common repertoire of effector proteins, thereby serving as conduits for the activation of diverse downstream signaling cascades. Ral GTPases are integral to several fundamental cellular processes, including gene expression, vesicle trafficking, cell migration, and invasion. Their functional output is highly context-dependent, allowing them to regulate distinct pathways according to cellular needs [18]. The spatiotemporal activity of Ral is precisely modulated by specific Ral GEFs and Ral GTPases, which transduce upstream signals to control the GTP-loading cycle.

A principal functional role of RalA and RalB lies in the regulation of exocytosis. Both isoforms are critical for the assembly and plasma membrane localization of the exocyst complex, an octameric protein machinery essential for the docking and fusion of secretory vesicles [19]. RalA primarily facilitates the recruitment and stabilization of the exocyst at the plasma membrane. In contrast, RalB, through its direct interaction with the exocyst component Sec5, activates the TANK-binding kinase 1 (TBK1), a kinase implicated in innate immune responses and vesicular trafficking [20]. Membrane association and full biological function of both RalA and RalB are dependent on lipid modification at a conserved cysteine residue within the C-terminal ‘CaaX’ motif (where C is cysteine, ‘aa’ are aliphatic residues, and X is typically serine, methionine, or leucine), a process essential for their proper subcellular targeting [19]. Notably, RalA and RalB can exhibit antagonistic biological roles despite their structural similarity. For instance, in KRAS-mutant bladder cancer cells, RalA suppresses cell motility while RalB promotes it [18]. Furthermore, RalA is known to activate key transcription factors such as Nuclear Factor-kappa B (NF-κB) and c-JUN, which are central mediators of inflammatory responses and tumor progression. Collectively, these findings underscore that the Ral pathway governs the expression of oncogenic and inflammatory gene programs, thereby playing a significant role in disease pathogenesis [21].

### 2.3. Signaling Pathways Involved in Ras-Ral Activation

Ras activation is principally mediated through two canonical signaling cascades: the mitogen-activated protein kinase (MAPK) pathway and the phosphoinositide 3-kinase (PI3K) pathway. Within the MAPK cascade, activation initiates with the binding of extracellular ligands, such as growth factors, to receptor tyrosine kinases (RTKs), leading to receptor dimerization and autophosphorylation [22]. This event facilitates the recruitment of adaptor proteins, including growth factor receptor-bound protein 2 (GRB2) and the guanine nucleotide exchange factor Son of Sevenless (SOS), which translocate to the plasma membrane and engage Ras [23]. SOS catalyzes the exchange of GDP for GTP on Ras, converting it to its active conformation. GTP-bound Ras subsequently recruits and activates RAF kinases at the membrane, a step involving the dissociation of inhibitory 14-3-3 proteins and the formation of a signaling complex with MAPK/ERK (MEK). This initiates the sequential phosphorylation cascade of MEK and ERK [24,25]. Phosphorylated ERK can, in turn, activate Ral guanine nucleotide exchange factors (RalGEFs), which promote the conversion of Ral-GDP to Ral-GTP, thereby linking mitogenic signaling to Ral-mediated cellular responses such as proliferation [25]). The quantitative kinetic factors controlling activation thresholds, signal amplitude, and duration in metabolic tissues are still poorly understood, despite documented molecular connections between Ras and RalGEFs. A supportive conceptual framework for comprehending how precise modulation of signaling kinetics may affect biological responses is provided by developments in stimulus-responsive hydrogel systems, which are capable of temporally controlled and feedback-regulated drug release [26,27]. In order to minimize chronic dysregulation and related metabolic dysfunction, it is suggested that treatment efforts should focus on restoring proper spatiotemporal activation patterns rather than just boosting or inhibiting system components. Oncogenic mutations in K-Ras and N-Ras, frequently at codons 12, 13, or 61, induce conformational changes that impair intrinsic and GAP-mediated GTP hydrolysis, resulting in constitutive Ras activation and sustained MAPK signaling [28].

The PI3K pathway is activated through a parallel mechanism initiated by RTK dimerization and phosphorylation. Activated Ras binds directly to the p110α catalytic subunit of PI3K, inducing an allosteric conformational change that stimulates its lipid kinase activity and promotes its association with the plasma membrane [29,30]. Concurrently, phosphorylated tyrosine residues on the activated RTK interact with the nSH2 domain of the p85 regulatory subunit of PI3K. This interaction relieves the inhibitory constraint exerted by p85 on p110α, further exposing the kinase domain for full activation [31]. Computational and structural studies have elucidated that the binding of oncogenic K-Ras4B to PI3Kα disrupts the autoinhibitory p85–p110 interface, inducing a rearrangement of the nSH2 domain and leading to constitutive PI3K activation. This aberrant signaling drives downstream processes critical for cell growth, proliferation, and survival [32].

In a complementary and often intersecting manner, Ras also activates Ral GTPases (RalA and RalB) primarily through Ras-binding RalGEFs, which facilitate GTP loading. Active, GTP-bound Ral engages a spectrum of downstream effectors, including Ral-binding protein 1 (RalBP1), components of the exocyst complex (e.g., Sec5 and Exo84), and phospholipase D1 (PLD1). These effectors coordinate essential cellular functions such as vesicle trafficking, cytoskeletal reorganization, and the regulation of cell proliferation and survival [19]. Notably, Ral activation is not exclusively dependent on Ras. Alternatively, Ras-independent mechanisms exist, involving other GEFs such as RalGPS1 and RalGPS2. These proteins contain pleckstrin homology (PH) domains and Src homology 3 (SH3)-binding motifs [33]. The PH domain of RalGPS2 binds phosphatidylinositol 4,5-bisphosphate (PIP2), anchoring the protein to the plasma membrane. In cellular models such as HEK293 cells, this membrane localization is associated with inducing membrane rippling and vesiculation, while its PxxP motif interacts with adaptor proteins like Grb2 and phospholipase C gamma (PLCγ), thereby linking Ral activation to broader tyrosine kinase signaling networks [33]. Furthermore, in Rat-2 fibroblasts, stimuli such as lysophosphatidic acid (LPA) and epidermal growth factor (EGF) can stimulate Ral activation through a PLC-mediated calcium signaling pathway. Here, PLC hydrolyzes PIP2 into inositol trisphosphate (IP3) and diacylglycerol (DAG). IP3 induces calcium (Ca^2+^) release from the endoplasmic reticulum, which subsequently contributes to Ral activation—a process that can proceed independently of Ras activity [34,35].

These findings underscore the multifaceted and context-dependent regulatory mechanisms governing the activation of Ral GTPases. The contrasting functions of Ras and Ral GTPases in glucose metabolism and insulin signaling are systematically compared in Table 1. While Ras activation is linked to insulin resistance, inflammation, and impaired GLUT4 trafficking, Ral signaling supports insulin secretion, vesicle docking, and glucose uptake. This dichotomy underscores the need for pathway-specific modulation in diabetes therapy, with Ras representing a target for inhibition and Ral for enhancement.

### 2.4. A Dual-Pathway Hypothesis: Rebalancing the Ras–Ral Axis for Diabetes Therapy

Building upon the established yet contrasting roles of Ras and Ral GTPases in metabolism, we propose a unifying hypothesis: The progression of T2DM involves a pathological imbalance in the Ras–Ral signaling axis, characterized by hyperactive Ras-driven pathways and insufficient Ral-mediated signaling. Pharmacological rebalancing of this axis—through concerted inhibition of Ras and enhancement of Ral activity—represents a novel and mechanistically grounded strategy to concurrently ameliorate insulin resistance and restore β-cell function. This hypothesis is predicated on several key observations synthesized from the literature:

#### 2.4.1. Divergent Metabolic Roles

Ras activation, particularly through the MAPK and PI3K pathways, is linked to insulin receptor substrate 1 (IRS-1) serine phosphorylation, chronic low-grade inflammation via NF-κB, and impaired GLUT4 trafficking, all hallmarks of insulin resistance [38,41,45]. In contrast, RalA activation promotes the exocyst-mediated docking of both insulin granules and GLUT4 vesicles, directly facilitating insulin secretion and glucose uptake [19].

#### 2.4.2. Shared Upstream Regulation, Divergent Outcomes

Both Ras and Ral are activated by common upstream stimuli (e.g., growth factors, nutrient overload), yet their downstream effects on metabolism are often antagonistic. This suggests that the cellular metabolic outcome is determined by the relative signaling flux through each branch.

#### 2.4.3. Evidence of Imbalance in Disease States

Persistent Ras activation combined with impaired Ral regulatory control causes context-dependent RalA signaling in obesity and T2DM. Under chronic stress, this signaling may shift from metabolic support to mitochondrial failure [46].

#### 2.4.4. Proof-of-Concept from Modulators

Preliminary support comes from interventions at either end of the axis. Ras inhibitors like F-FTS improve insulin sensitivity and reduce inflammation [37], while genetic enhancement of Ral signaling improves glucose tolerance [39]. However, these approaches have been studied in isolation. The proposed dual-pathway strategy offers a synergistic advantage over single-target approaches. Isolated Ras inhibition may alleviate insulin resistance but does not directly augment the secretory capacity of failing β-cells. Conversely, Ral enhancement alone may boost insulin secretion but not fully counteract the pervasive inflammatory and inhibitory signals driven by hyperactive Ras. Simultaneous modulation targets both the cause (Ras-driven dysfunction) and the consequence (insufficient Ral-mediated compensation) of the metabolic defect.

This hypothesis generates several testable predictions for future research:

Prediction 1: Combination therapy using a Ras inhibitor (e.g., a farnesyltransferase inhibitor (FTI) or a specific KRAS modulator) and a Ral enhancer will show superior efficacy in improving glycemic control and preserving β-cell mass in animal models of T2DM compared to either agent alone.

Prediction 2: Tissue-specific knockout models will reveal that the metabolic benefits of Ras inhibition are primarily mediated in the liver and muscle, while the benefits of Ral activation are strongest in pancreatic islets and adipose tissue.

Prediction 3: Biomarkers of Ras pathway activity (e.g., p-ERK, specific phospho-IRS-1 epitopes) will correlate with insulin resistance, while biomarkers of Ral/exocyst activity will correlate with preserved insulin secretion in human cohorts.

**Figure 1:**
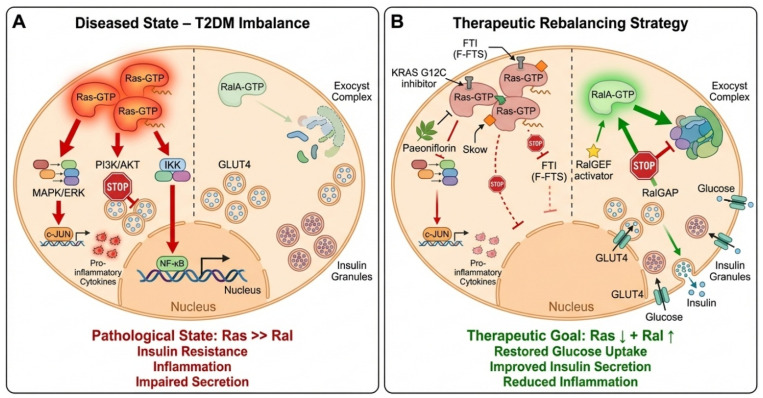
This figure illustrates the central hypothesis of this review. (**A**) In Type 2 DM, a signaling imbalance favors hyperactive Ras pathways that promote inflammation and inhibit metabolic processes, while Ral-mediated exocytotic trafficking is insufficient. (**B**) The proposed therapeutic strategy involves dual modulation: pharmacologically inhibiting Ras (via farnesyltransferase inhibitors, allele-specific inhibitors, or natural compounds) while enhancing Ral signaling (via RalGEF agonists or RalGAP inhibitors). This coordinated rebalancing aims to restore GLUT4 translocation, insulin secretion, and metabolic homeostasis.

By framing the review around this central hypothesis, we move beyond cataloging associations to proposing a concrete, actionable therapeutic paradigm. Validating this hypothesis will require interdisciplinary efforts combining chemical biology to develop selective Ral activators, systems pharmacology to test combination regimens, and translational studies to identify patient subgroups most likely to benefit from this targeted approach. This dual-pathway hypothesis is illustrated schematically in Figure 1. In the diabetic state (Figure 1A), Ras hyperactivity drives inflammation and insulin resistance, while Ral-mediated vesicular trafficking is deficient. The proposed therapeutic intervention (Figure 1B) combines Ras inhibition with Ral enhancement to rebalance the axis, thereby restoring insulin sensitivity, promoting glucose uptake, and preserving β-cell secretory function. This model encapsulates our central thesis: that coordinated modulation of the Ras–Ral signaling equilibrium represents a novel and mechanistically grounded strategy for the treatment of T2DM.

It is crucial to stress that, despite its conceptual appeal, the suggested dual-pathway approach is currently predicated on the integration of results from research that has mostly looked at Ras and Ral signaling separately. As of now, there is no concrete experimental proof that concurrent Ras suppression and Ral regulation provide better therapeutic outcomes than single-pathway treatments. As a result, this framework should be regarded as a hypothesis-generating model that has to be rigorously validated in both preclinical and translational contexts using combinatorial experimental techniques. Before its therapeutic potential can be completely proven, it will be necessary to clarify the effectiveness, safety, and context-specific results of such dual modulation.

## 3. The Role of Ras-Ral Axis in Diabetes

Glucose homeostasis is a tightly regulated physiological process fundamentally dependent on glucose-stimulated insulin secretion (GSIS) by pancreatic β-cells. In response to hyperglycemia, β-cells synthesize and secrete insulin to restore normoglycemia. However, insulin resistance—a pathological state driven by genetic susceptibility, chronic inflammation, lipotoxicity, and prolonged hyperglycemia—constitutes a central hallmark of T2DM [47]. Lipotoxicity, frequently associated with obesity and metabolic imbalance, results from excessive lipid accumulation in non-adipose tissues and significantly exacerbates insulin resistance. Obesity has been demonstrated to amplify Ras signaling in adipose tissue, promoting adipocyte hypertrophy and macrophage infiltration, which collectively foster a state of chronic inflammation and worsen insulin sensitivity [38].

**Figure 2:**
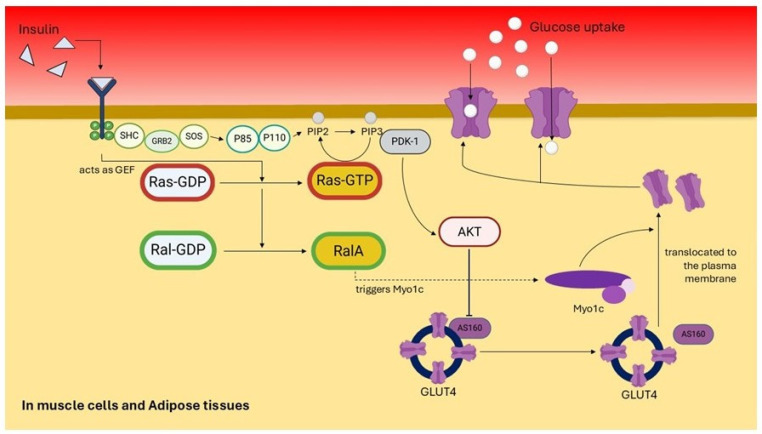
Schematic representation of insulin-stimulated glucose uptake via Ras and RalA signaling pathways in muscle and adipose tissues. Upon insulin binding to its receptor, a cascade is initiated involving SHC, GRB2, and SOS, which acts as a guanine nucleotide exchange factor (GEF) to activate Ras by converting Ras-GDP to Ras-GTP. Ras-GTP subsequently activates PI3K (P85/P110 complex), leading to the conversion of PIP2 to PIP3. PIP3 recruits and activates PDK1, which phosphorylates and activates AKT. Activated AKT promotes GLUT4 vesicle translocation to the plasma membrane via AS160 inhibition and Myo1c activation, enhancing glucose uptake. Concurrently, Ras-GTP activates RalA through RalGEFs, facilitating GLUT4 vesicle trafficking and fusion with the membrane. This coordinated signaling network ensures efficient glucose uptake in response to insulin stimulation.

Pharmacological inhibition of Ras has emerged as a promising strategy to counteract these metabolic disturbances. Administration of F-FTS, a potent Ras inhibitor, has been shown to reduce serum insulin levels while enhancing peripheral glucose uptake in murine models. Furthermore, Ras inhibition downregulates the Nuclear Factor-kappa B (NF-κB) pathway, thereby attenuating obesity-associated inflammation in high-fat diet (HFD)-fed mice [37]. Similarly, dysregulated H-Ras activity has been implicated in impairing insulin sensitivity, an effect that can be ameliorated by treatment with agents such as paeoniflorin [14]. Upon insulin stimulation, the translocation of the glucose transporter GLUT4 to the plasma membrane, a process mediated by motor proteins including Myo1c and the small GTPase Rac1, is essential for facilitating glucose uptake in skeletal muscle and adipose tissue [42]. Ral GTPases, particularly RalA, contribute to this process via the PI3K/Akt signaling axis, where they promote GLUT4-containing vesicle exocytosis and subsequent cellular glucose entry [48]. Conversely, phosphatase and tensin homolog (PTEN), a lipid phosphatase often modulated by Ras activity, negatively regulates this pathway by dephosphorylating phosphatidylinositol (3,4,5)-trisphosphate (PIP3) to phosphatidylinositol (4,5)-bisphosphate (PIP2), thereby antagonizing Akt activation and potentially diminishing glucose uptake [49]. Figure 2 provides a mechanistic overview of insulin-stimulated glucose uptake mediated by Ras and RalA in muscle and adipose tissue. Upon insulin binding, Ras activates the PI3K/AKT pathway, promoting GLUT4 vesicle translocation. Simultaneously, Ras activates RalA via RalGEFs, which facilitates vesicle docking and fusion through the exocyst complex. This coordinated signaling ensures efficient glucose uptake, highlighting the complementary roles of Ras and RalA in insulin action and their disruption in insulin-resistant states.

While Ral GTPases appears to facilitate glucose absorption and potentiate insulin secretion, aberrant Ras signaling often exerts inhibitory effects under pathological conditions. Supporting this dichotomy, the statin drug atorvastatin has been reported to impair insulin synthesis in β-cells through activation of the Ras pathway, whereas Ras inhibitors like F-FTS confer β-cell protection [37]. In contrast, RalA plays a pivotal and positive role in insulin granule trafficking and docking by engaging in the exocyst complex, a key RalA effector. This complex facilitates the tethering and fusion of insulin-containing secretory vesicles with the plasma membrane, a critical step for GSIS [39].

**Figure 3:**
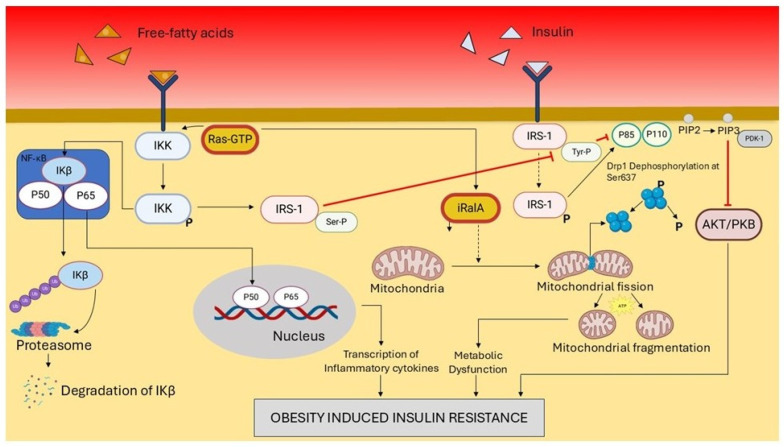
Mechanistic illustration of obesity-induced insulin resistance via Ras and RalA-mediated pathways. Elevated levels of free fatty acids and insulin contribute to the activation of Ras-GTP, which subsequently stimulates IKK activation. IKK phosphorylates IκB, leading to its ubiquitination and degradation via the proteasome, allowing NF-κB (P50/P65) to translocate to the nucleus and induce transcription of proinflammatory cytokines. Simultaneously, insulin activates the PI3K/AKT pathway through IRS-1 phosphorylation, promoting glucose uptake and mitochondrial integrity. However, Ras and iRalA (inactive RalA) interfere with insulin signaling by promoting serine phosphorylation of IRS-1, impairing downstream AKT activation. This dysregulation leads to mitochondrial fission and fragmentation due to reduced Drp1 Ser637 dephosphorylation, contributing to metabolic dysfunction. Collectively, these molecular events culminate in chronic inflammation and mitochondrial stress, promoting obesity-induced insulin resistance.

The pathogenic influence of the Ras–Ral axis extends to developmental and tissue-specific metabolic contexts. In maternal obesity, elevated Ras activity within the placental microenvironment contributes to fetal insulin resistance and impaired metabolic programming by exacerbating local inflammation and disrupting nutrient transport mechanisms [50]. Additionally, RalA activation has been mechanistically linked to obesity-induced mitochondrial fragmentation in white adipocytes, a process that promotes insulin resistance and reduces mitochondrial oxidative capacity. Obesity elevates the activity of a distinct, inactive RalA (iRalA) pool, which promotes the dephosphorylation of dynamin-related protein 1 (Drp1) at serine 637. This post-translational modification triggers excessive mitochondrial fission, contributing significantly to systemic metabolic dysfunction [46]. The pathological interplay between Ras, RalA, and mitochondrial dysfunction in obesity-induced insulin resistance is depicted in Figure 3.

**Figure 4:**
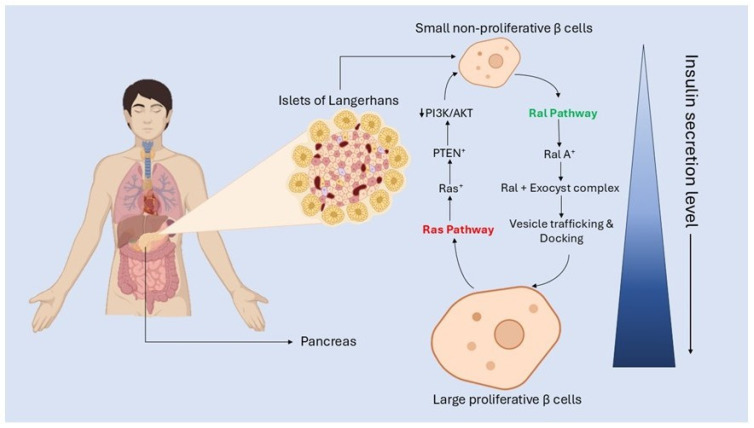
Differential roles of Ras and Ral signaling pathways in β-cell proliferation and insulin secretion. The image illustrates the localization of the pancreas and the Islets of Langerhans within the human body. Within the islets, β cells exhibit functional heterogeneity based on proliferative and secretory capacity. The Ras pathway (red) promotes β-cell proliferation but is associated with upregulation of PTEN, which negatively regulates the PI3K/AKT signaling axis, leading to reduced insulin secretion. In contrast, the Ral pathway (green) enhances insulin secretion by activating RalA, which forms a complex with the exocyst, facilitating insulin vesicle trafficking and docking at the plasma membrane. This results in efficient insulin release from large, metabolically active β cells, whereas Ras activation supports smaller, less secretory β cells. A gradient indicates the inverse correlation between proliferative state and insulin secretion efficiency.

Hyperactive Ras promotes inflammation via NF-κB activation, while inactive RalA (iRalA) drives mitochondrial fragmentation through Drp1 dephosphorylation. These events collectively impair insulin signaling and glucose metabolism, illustrating how Ras–Ral imbalance exacerbates metabolic stress and insulin resistance in obesity. These collective insights underscore the contrasting and context-dependent roles of Ras and Ral GTPases in the pathogenesis of diabetes. Ras activation is frequently associated with β-cell impairment and the progression of insulin resistance, whereas Ral activity supports crucial insulin secretory processes and facilitates cellular glucose uptake, notably through mechanisms such as GLUT4 translocation. Intriguingly, both Ras and Ral signaling are upregulated in the setting of obesity, reflecting their shared regulatory inputs yet divergent functional outcomes in metabolic disease. This duality highlights the therapeutic potential of interventions that selectively modulate Ras and Ral activity to restore metabolic equilibrium in diabetes. The differential roles of Ras and Ral in β-cell function are schematically summarized in Figure 4.

Ras activation promotes β-cell proliferation but is associated with increased PTEN expression, which inhibits PI3K/AKT signaling and impairs insulin secretion. In contrast, RalA activation facilitates insulin vesicle exocytosis via the exocyst complex, enhancing insulin release. This model illustrates the inverse relationship between proliferative signaling and secretory efficiency in β-cells, underscoring the therapeutic potential of rebalancing Ras–Ral signaling to preserve both β-cell mass and function. The experimental foundation linking Ras–Ral dysregulation to metabolic dysfunction is compiled in Table 2. This table synthesizes evidence from cellular, animal, and human studies, demonstrating how Ras hyperactivation contributes to insulin resistance and β-cell failure, while Ral activation supports glucose uptake and insulin secretion. Together, these findings validate the Ras–Ral axis as a pivotal regulator of metabolic homeostasis and a viable target for therapeutic intervention.

### Implications of Ras-Ral Axis Dysregulation in Diabetes

The Ras–Ral signaling axis constitutes a critical regulatory node in the integration of insulin action, inflammatory responses, and systemic glucose metabolism. Its dysregulation is intimately associated with the initiation and progression of T2DM. Lifestyle determinants, including poor dietary patterns and physical inactivity, induce a state of chronic metabolic stress that promotes the pathological overactivation of Ras signaling. This hyperactivity subsequently impairs the physiological, insulin-sensitizing functions mediated by Ral GTPases, such as the potentiation of insulin secretion and the facilitation of GLUT4 vesicle translocation [51]. Within the context of obesity—a primary risk factor for T2DM—elevated circulating free fatty acids and expanded adipose mass serve as potent activators of Ras-mediated pathways, including the MAPK and PI3K cascades. This sustained activation disrupts proximal insulin receptor signaling, creating a state of cellular insulin resistance. Chronic overnutrition perpetuates this condition, maintaining Ras in a constitutively active state that impedes normal GLUT4 trafficking, diminishes cellular glucose uptake, and fundamentally contributes to the establishment of systemic insulin resistance [41]. In contrast to the detrimental effects of sustained Ras activation, Ral GTPases support insulin functionality by orchestrating cytoskeletal architecture and regulating vesicular trafficking pathways. These functions are indispensable for the exocytosis of insulin granules from pancreatic β-cells and the insertion of GLUT4 transporters into the plasma membrane of insulin-sensitive tissues. Moreover, the obesity-associated chronic inflammatory milieu amplifies Ras signaling through pro-inflammatory mediators such as tumor necrosis factor-alpha (TNF-α) and interleukin-6 (IL-6). These cytokines activate the NF-κB pathway, which in turn reinforces Ras activity, creating a vicious cycle that further impairs insulin sensitivity and perpetuates metabolic dysfunction [38]. A study conducted by Chen and its team has stated that κB-Ras stabilizes IκBβ by forming a ternary complex with NF-κB, thereby inhibiting IKKβ-mediated phosphorylation and preventing its degradation. Consequently, complete IκBβ degradation requires additional signal inputs beyond IKK activation alone [52]. κB-Ras functions as a dual tumor suppressor by concurrently restraining NF-κB–mediated inflammation/cell survival and Ral-driven proliferation. Loss of κB-Ras amplifies both pathways, promoting tumor growth, whereas its restoration suppresses anchorage-independent proliferation [53].

The divergent and often antagonistic roles of Ras and Ral within the same signaling network underscore the necessity for precise and balanced regulation of this axis. The pathological shift towards Ras hyperactivation and relative Ral insufficiency disrupts this equilibrium, directly contributing to the core defects of T2DM. Consequently, therapeutic strategies that simultaneously inhibit pathological Ras signaling and enhance or preserve beneficial Ral activity may represent a novel and rational approach to restoring insulin responsiveness and achieving more effective long-term management of T2DM.

## 4. Potential Therapeutic Approaches Targeting the Ras-Ral Axis in Diabetes

The development of therapeutic strategies that concurrently suppress pathological Ras overactivation and enhance beneficial Ral activity presents a promising paradigm for restoring effective insulin secretion and re-establishing metabolic homeostasis in diabetes. This dual-modulation approach is designed to rebalance the signaling equilibrium between the Ras-driven pathways that promote insulin resistance and inflammation, and the Ral-mediated processes that support insulin granule exocytosis and GLUT4 translocation. However, directly targeting Ras proteins with conventional small-molecule inhibitors has historically been challenging, leading to their characterization as “undruggable.” This difficulty stems primarily from the absence of well-defined, deep binding pockets on the Ras surface suitable for high-affinity drug interaction [54]. Contemporary therapeutic innovation has therefore shifted focus towards alternative strategies. These include targeting essential post-translational modifications of Ras—such as farnesylation—and disrupting critical protein–protein interactions between Ras and its downstream effector proteins to abrogate oncogenic and metabolic signaling cascades [55]. In parallel, enhancing the activity of Ral GTPases, particularly in contexts governing vesicle trafficking and insulin exocytosis, could serve as a compensatory mechanism to bolster insulin signaling. Collectively, this combinatorial pharmacological strategy, aimed at fine-tuning the Ras–Ral axis, may unlock novel avenues for mitigating insulin resistance and advancing precision medicine for T2DM.

### 4.1. Direct and Indirect Pharmacological Inhibition of Ras

Therapeutic targeting of Ras has evolved to encompass strategies that either directly modulate its conformational states or indirectly inhibit its activation and downstream signaling. Given the historical challenge of targeting Ras due to its smooth surface and lack of deep hydrophobic pockets, innovative approaches have focused on stabilizing specific inactive conformations, disrupting effector interactions, and inhibiting critical post-translational modifications.

#### 4.1.1. Small-Molecule Inhibitors Targeting Ras Conformational States

Ras GTPases exist in dynamic conformational equilibria, and their functional output is governed by transitions between these states. Small molecules that stabilize conformations with reduced affinity for effector proteins can effectively disrupt downstream signaling. Notable examples include metal-chelating complexes such as Zn^2+^-cyclen and Cu^2+^-cyclen, which target the nucleotide-binding pocket of Ras [56]. Zn^2+^-cyclen binds directly within this site, interacting with GTP and stabilizing Ras in a conformation that prevents productive engagement with effectors like Raf [57]. Similarly, Cu^2+^-cyclen occupies the nucleotide-binding pocket, shifting the conformational equilibrium toward the inactive GDP-bound state and thereby inhibiting Ras-effector interactions [56].

#### 4.1.2. Inhibitors Targeting Ras Effector Binding and Activation

An alternative strategy involves disrupting the interface between Ras and its downstream effectors. Sulindac sulfide, a non-steroidal anti-inflammatory drug (NSAID), exemplifies this approach by interfering with Ras–GTP binding to c-Raf-1 and impairing GEF-catalyzed nucleotide exchange [58]. It binds non-covalently to Ras, accelerating GTP hydrolysis via p120GAP while inhibiting nucleotide exchange catalyzed by CDC25, thereby suppressing Ras-induced oncogenic transformation in fibroblasts without broadly affecting normal proliferation [59]. However, its clinical utility remains limited due to off-target effects and lack of specificity in vivo [60]. Fragment-based approaches have also identified inhibitors that bind at the Ras–SOS interface, such as compounds targeting “Site A” (a hydrophobic pocket on SOS) or “Site B” (the Ras–SOS1 interface), which stabilize inactive complexes and prevent GTP reloading [61].

#### 4.1.3. Inhibition of Ras Membrane Localization and Downstream Signaling

Farnesylation, the attachment of a 15-carbon farnesyl isoprenoid to a C-terminal cysteine, is essential for Ras membrane localization and full biological activity. FTIs block this modification, thereby mislocalizing Ras and inhibiting its function. F-FTS is a potent Ras inhibitor that has demonstrated efficacy in metabolic disease models, reducing serum insulin and glucose levels while suppressing NF-κB-mediated inflammation [37]. However, the clinical application of FTIs in cancers with KRAS or NRAS mutations has been limited due to alternative prenylation by geranylgeranyltransferase. To overcome this, combination therapies pairing FTIs with KRAS-G12C–specific inhibitors have shown enhanced antitumor effects [62]. Salirasib (S-trans, transfarnesylthiosalicylic acid), a synthetic Ras inhibitor that mimics the farnesyl group, competes with Ras for membrane anchorage. While effective in HRAS-mutated bladder cancer models, its in vivo efficacy is modest [63].

#### 4.1.4. Allele-Specific and Natural Compound Inhibitors

The advent of allele-specific inhibitors has revolutionized direct Ras targeting. Beyond synthetic molecules, natural compounds have also shown promise in modulating Ras-related pathways in diabetes. Paeoniflorin, a monoterpenoid glycoside from *Paeonia* species, downregulates HRAS expression in a dose-dependent manner, protecting pancreatic β-cells from high glucose-induced apoptosis [14]. Similarly, glycyrrhetinic acid (GA), a bioactive component of liquorice, enhances insulin sensitivity by modulating the Ras/MAPK pathway, inhibiting IRS-1 phosphorylation at Ser307, and promoting glucose uptake [64].

### 4.2. Enhancement of Ral Activity as a Therapeutic Strategy

In contrast to Ras inhibition, enhancing Ral GTPase activity offers a complementary therapeutic approach aimed at augmenting insulin secretion and GLUT4 translocation. Ral activation is primarily mediated by guanine nucleotide exchange factors (GEFs), notably Ral guanine nucleotide dissociation stimulator (RalGDS), which is directly engaged by active Ras, thereby coupling Ral activation to upstream mitogenic and metabolic signals [65]. RalGDS catalyzes the exchange of GDP for GTP on RalA and RalB, promoting their active conformations. The effector protein RLIP76 (RalA-binding protein 1) binds specifically to GTP-bound RalA, serving as a critical node that integrates Ral signaling with cytoskeletal regulation and vesicular transport. Given the central role of RalA in insulin granule exocytosis and GLUT4 vesicle docking, pharmacological strategies designed to enhance Ral activity—through direct GEF activation or stabilization of the active Ral conformation—represent a novel avenue for improving glucose homeostasis in diabetes.

### 4.3. Ras–Ral Axis Modulation Within Current Antidiabetic Therapies

Recent advancements in the management of T2DM have been profoundly influenced by incretin-based therapies, especially glucagon-like peptide-1 (GLP-1) receptor agonists, which enhance glycemic control through the augmentation of glucose-dependent insulin secretion, inhibition of glucagon release, prolongation of gastric emptying, and facilitation of weight reduction [66]. Agents such as semaglutide and liraglutide have exhibited significant efficacy in lowering glycated hemoglobin HbA1c levels and mitigating cardiovascular risk. Nevertheless, these strategies predominantly focus on downstream metabolic control and do not directly confront the upstream signaling dysregulation that leads to insulin resistance and β-cell failure [67]. The suggested Ras–Ral rebalancing technique seeks to intervene at a more proximal level of intracellular signaling by concurrently inhibiting Ras-driven inflammatory and insulin-resistant pathways while promoting Ral-mediated vesicular trafficking and insulin production. This dual modulation provides a supplementary mechanism that may improve or maintain the effectiveness of current medications, especially in individuals with progressive β-cell dysfunction or chronic inflammatory conditions [37].

### 4.4. Integrated Modulation of the Ras–Ral Axis

The Ras and Ral GTPases play opposing yet interconnected roles in diabetes pathophysiology. Ras activation promotes insulin resistance via MAPK/PI3K hyperactivation and inflammatory signaling, whereas Ral activity supports insulin secretion and glucose uptake. Therapeutic success may therefore depend on a dual-regimen strategy: selectively inhibiting pathogenic Ras signaling while potentiating beneficial Ral-mediated processes. Advances in direct Ras inhibition (e.g., allele-specific inhibitors, FTIs) and emerging strategies for Ral enhancement provide a robust foundation for targeted intervention. Future research should prioritize the development of isoform-selective modulators, evaluate combination therapies in relevant diabetic models, and elucidate the precise molecular crosstalk that defines the Ras–Ral equilibrium in metabolic tissues. A range of pharmacological agents targeting the Ras–Ral axis—including Ras inhibitors and proposed Ral enhancers—are summarized in Table 3. This catalogue highlights both established and emerging compounds, their mechanisms, preclinical evidence, and developmental stages. The table illustrates the growing arsenal for Ras inhibition and identifies key gaps in the development of Ral-targeted therapeutics, guiding future drug discovery efforts.

## 5. Ral Enhancers: A Therapeutic Avenue for Restoring Insulin Sensitivity and Secretion

Given the protective role of Ral GTPases in glucose metabolism, strategies aimed at enhancing Ral activity present a compelling therapeutic frontier for diabetes management. In contrast to the pathogenic overactivation of Ras, potentiation of Ral signaling—particularly of RalA—can directly address core defects in T2DM by augmenting insulin secretion from pancreatic β-cells and facilitating GLUT4-mediated glucose uptake in peripheral tissues. Therapeutic enhancement of Ral function may be achieved through several mechanistic approaches: direct pharmacological activation of Ral GTPases, modulation of upstream Ral-specific guanine nucleotide exchange factors (RalGEFs), or stabilization of active Ral–effector complexes [65]. Beyond Ras-dependent activation, other RalGEFs such as RalGPS1 and RalGPS2 can activate Ral in a Ras-independent manner, often through phospholipase C (PLC) and calcium-mediated pathways [33,34]. This multiplicity of activation mechanisms provides several potential nodes for pharmacological intervention. For instance, small molecules that mimic the activating function of RalGDS or allosterically enhance the activity of endogenous RalGEFs could selectively boost Ral-GTP levels in insulin-sensitive tissues.

Downstream of activation, RalA exerts its insulin-sensitizing effects largely through the exocyst complex, a multi-subunit tethering machinery essential for the docking and fusion of GLUT4 vesicles and insulin granules with the plasma membrane. RalA binds directly to exocyst components Sec5 and Exo84, promoting assembly and membrane localization of the complex [19]. Therefore, compounds that stabilize the RalA–exocyst interaction or enhance exocyst assembly could functionally mimic Ral activation even without directly increasing Ral-GTP levels. To date, few direct Ral activators have been reported, but early-stage small molecules such as pyrido-pyrimidine derivatives have been identified in screens for Ral pathway activators in cancer contexts. Repurposing or optimizing such compounds for metabolic indications represents an underexplored opportunity.

An alternative strategy involves inhibiting the negative regulators of Ral, such as Ral GTPase-activating proteins (RalGAPs). The RalGAP complex, composed of RalGAPα and RalGAPβ subunits, accelerates GTP hydrolysis on RalA and RalB, terminating their signal. Genetic ablation of RalGAPβ in mice results in enhanced RalA activity, improved glucose tolerance, and increased insulin sensitivity, highlighting RalGAPs as valid therapeutic targets for diabetes [39]. Small-molecule inhibitors of RalGAP activity could therefore sustain endogenous Ral signaling, offering a parallel approach to GEF activators.

In the context of diabetic complications, Ral activation may also confer benefits beyond glycemic control. For example, in endothelial cells, RalA signaling promotes nitric oxide synthase (eNOS) activation and improves vascular function, suggesting that Ral enhancers could mitigate diabetes-associated cardiovascular risk. Furthermore, by counteracting the pro-inflammatory and pro-apoptotic signals often amplified by hyperactive Ras, enhanced Ral activity may protect pancreatic β-cells from glucolipotoxicity and preserve functional β-cell mass.

Despite this compelling rationale, the development of clinically viable Ral enhancers remains in its infancy. Key challenges include achieving tissue selectivity—particularly in pancreatic β-cells, muscle, and adipose tissue—and avoiding potential oncogenic side effects given Ral’s role in tumor progression. In this context, similarities can be taken with advancements in stimuli-responsive nanocomposite hydrogels, designed for spatiotemporally regulated, cascade-based activation in response to microenvironmental cues, including pH, reactive oxygen species, and glucose concentrations. These systems provide coordinated, multi-step regulation of cellular processes, encompassing vesicle-like transport and precise cargo release, emulating exocyst-mediated dynamics [68]. Analogously, embracing modular and environmentally adaptive design principles may offer a conceptual framework for the development of next-generation Ral activators, facilitating precise, context-specific regulation of exocytic pathways and enhancing therapeutic targeting in intricate pathological conditions such as diabetes-related impaired wound healing.

Future research must prioritize the identification and optimization of selective RalA activators, evaluate their efficacy and safety in preclinical models of diabetes and insulin resistance, and delineate the precise downstream effectors responsible for their metabolic benefits. Success in this endeavor would not only validate the Ral pathway as a druggable target for diabetes but also provide a novel class of therapeutics that address both insulin deficiency and resistance through a single molecular target.

## 6. Future Directions and Translational Challenges

The therapeutic potential of the Ras–Ral axis in diabetes, while compelling, is accompanied by significant translational hurdles that must be systematically addressed to advance this paradigm from hypothesis to clinical application. The following sections delineate the principal challenges and essential research priorities for the field.

### 6.1. Achieving Specificity and Mitigating Oncogenic Risk

The paramount challenge in targeting the Ras–Ral network lies in achieving sufficient pharmacological specificity for metabolic tissues while circumventing potential oncogenic consequences. Both Ras and Ral are established drivers of cellular proliferation and are frequently dysregulated in cancer. Therefore, systemic modulation of these pathways carries an intrinsic risk of promoting tumorigenesis or accelerating the growth of pre-existing malignancies. Overcoming this barrier necessitates a multi-pronged strategy.

#### 6.1.1. Development of Tissue-Selective Delivery Systems

Future therapeutic agents should be engineered for selective delivery to key metabolic organs—namely, pancreatic islets, liver, skeletal muscle, and adipose tissue—while minimizing exposure to proliferation-sensitive epithelia. This could be achieved through the development of nanoparticle carriers decorated with tissue-homing ligands, the design of antibody-drug conjugates targeting cell-surface proteins abundant in metabolic tissues, or the application of gene therapy vectors driven by tissue-specific promoters.

#### 6.1.2. Pursuit of Pathway-Selective Modulation

A more nuanced approach involves developing agents that disrupt specific effector interactions within the Ras–Ral cascade that are preferentially linked to metabolic dysfunction, while sparing those critical for normal growth and homeostasis. For instance, small molecules could be designed to selectively inhibit the interaction between Ras and the p110α subunit of PI3K in hepatocytes, a key node in hepatic insulin resistance, without affecting Ras–RAF binding essential for epithelial cell survival. Similarly, the design of Ral activators could focus on stabilizing RalA interactions with the exocyst complex in β-cells, while avoiding broad activation of proliferative transcription factors like c-JUN or NF-κB.

#### 6.1.3. Exploitation of Context-Dependent Signaling (Biased Modulation)

The functional output of Ral GTPases is highly context-dependent, dictated by the availability of specific effector proteins in different cell types. This dichotomy presents an opportunity for “biased” pharmacological modulation. In pancreatic β-cells and adipocytes, RalA’s primary metabolic role is mediated through the exocyst. In contrast, in certain cancers, Ral signaling may proceed through alternative effectors like RalBP1 to drive invasion. Therefore, drug discovery efforts should aim to develop Ral modulators that allosterically favor exocyst engagement over other effector interactions, thereby achieving a metabolic benefit without a concomitant pro-oncogenic effect.

### 6.2. Defining the Human Disease Phenotype and Biomarker Discovery

A critical translational step is to identify the subset of patients with diabetes or prediabetes whose pathophysiology is characterized by a demonstrable “Ras–Ral axis imbalance.” This precision medicine approach requires the discovery and validation of robust biomarkers.

#### 6.2.1. Identification of Circulating Biomarkers

Research should aim to identify proteins, metabolites, or microRNAs in serum or plasma that reflect the activity state of Ras or Ral pathways. Candidates may include specific phospho-proteins released from tissues, inflammatory cytokines downstream of Ras–NF-κB signaling, or extracellular vesicles carrying Ral pathway components.

#### 6.2.2. Development of Functional Cellular Assays

Functional biomarkers could be derived from accessible tissues, such as peripheral blood mononuclear cells (PBMCs) or cultured fibroblasts from skin biopsies. Assays measuring Ras/GTP loading, Ral-GTP levels, or exocyst assembly in response to standardized stimuli could provide a dynamic readout of pathway activity relevant to metabolic status.

#### 6.2.3. Establishment of Genetic and Transcriptomic Signatures

Large-scale genomic and transcriptomic analyses of well-phenotyped human cohorts are needed to identify genetic polymorphisms (e.g., molecular variants located in *RALA*, *RALGDS*, or *RASA* genes) or gene expression signatures within the Ras–Ral network that correlate with disease susceptibility, progression, or differential response to existing therapies like metformin or thiazolidinediones.

### 6.3. Advancing the Pharmacology of Ral Enhancement

The development of Ral-targeted therapeutics lags significantly behind that of Ras inhibitors, creating a major gap in the proposed dual-modulation strategy. Recent developments in polymeric controlled-release platforms offer a crucial translational framework for putting intricate signaling-based treatment approaches into practice. Injectable hydrogels, polymeric micelles, and poly (lactic-co-glycolic acid) (PLGA) nanoparticles are examples of biodegradable systems that have shown promise in delivering metabolic agents in a sustained, tissue-targeted manner while reducing systemic exposure and off-target effects [36]. Polymer-based insulin delivery systems enable controlled and stimuli-responsive release through mechanisms such as diffusion, swelling, and matrix degradation, improving glycemic regulation. These methods facilitate the advancement of non-invasive delivery systems and improve patient adherence by reducing the drawbacks of traditional subcutaneous injection [69]. Through regulated and site-specific release, these platforms have already demonstrated promise in enhancing the pharmacokinetics and efficacy of insulin, incretin-based treatments, and anti-inflammatory drugs in the context of diabetes [43]. These developments are especially important to the hypothesized Ras–Ral dual-modulation approach, where accurate spatiotemporal regulation of pathway activity is essential. Closing this gap requires a dedicated research agenda, which can be addressed from the following 4 perspectives.

#### 6.3.1. Implementation of Targeted High-Throughput Screening

Cell-based high-throughput screening platforms should be established to identify novel small-molecule Ral activators. Ideal screening reporters include Ral-GTP biosensors (e.g., based on fluorescence resonance energy transfer), reporters of exocyst complex assembly or activity, or phenotypic assays measuring GLUT4 translocation or insulin granule exocytosis [70]. High-throughput screening (HTS) methodologies utilizing FRET- or BRET-based Ral-GTP biosensors permit real-time monitoring of RalA and RalB activation in live cells, hence aiding in the identification of small-molecule modulators [71].

#### 6.3.2. Application of Structural Biology for Rational Drug Design

The rational design of high-affinity, selective Ral modulators is currently hampered by a lack of detailed structural information for key complexes. Priority should be given to solving high-resolution crystal or cryogenic electron microscopy (Cryo-EM) structures of RalA and RalB in complex with their metabolic GEFs (e.g., RalGDS) and effectors (e.g., the Exo84 subunit of the exocyst). These structures would reveal novel, druggable pockets for allosteric modulation [20].

#### 6.3.3. Rigorous Preclinical Validation in Relevant Disease Models

Promising candidate compounds must be rigorously evaluated in vivo using complementary rodent models of T2DM, such as diet-induced obese mice, leptin receptor-deficient (*db*/*db*) mice, and Zucker diabetic fatty (ZDF) rats [72]. Studies should assess not only acute effects on glycemia but also long-term outcomes on insulin sensitivity, β-cell function and mass, and tissue-specific signaling [73]. Beyond acute glycemic control, studies should evaluate long-term outcomes such as insulin sensitivity, β-cell function and mass, and systemic metabolic remodeling, which are central to T2DM progression [74].

### 6.4. Systematic Evaluation of Combination Therapy Regimens

The core therapeutic hypothesis advocates for the concurrent inhibition of Ras and enhancement of Ral. Testing this paradigm necessitates systematic preclinical combination studies.

#### 6.4.1. Assessment of Pharmacodynamic Synergy and Determination of Safety and Tolerability Profiles

Initial studies should evaluate whether co-administration of a Ras inhibitor (e.g., F-FTS) with a Ral pathway enhancer yields synergistic or additive improvements in glucose tolerance, insulin sensitivity, and insulin secretion in animal models, compared to monotherapy. Comprehensive toxicology studies must be conducted to establish the safety profile of such combinations, with particular attention to specific organ function (e.g., liver, kidney), potential for hypoglycemia, and long-term monitoring for tumor development in sensitive models.

#### 6.4.2. Optimization of Therapeutic Dosing and Scheduling

Research should explore whether continuous, intermittent, or sequential administration of the two classes of agents yields, namely Ras pathway modulators and Ral activators, yields the optimal therapeutic index. the optimal therapeutic index. Pharmacokinetic and pharmacodynamic modeling will be essential to guide the dosing of these dosing regimens. Given that Ras functions upstream of RalA/RalB, sequential or temporally controlled dosing may enhance pathway specificity while minimizing off-target effects associated with sustained Ras activation. Pharmacokinetic (PK) and pharmacodynamic (PD) modeling will be essential to define drug exposure–response relationships, optimize dosing intervals, and predict synergistic or antagonistic interactions [75].

### 6.5. Exploration of Non-Canonical and Tissue-Specific Signaling Roles

Gaining a complete understanding of the Ras–Ral axis in metabolism requires looking beyond the canonical pathways, which are delineated in the following 3 aspects.

#### 6.5.1. Elucidation of Isoform-Specific Functions

The distinct, and sometimes opposing, roles of RalA and RalB in different metabolic tissues (e.g., adipose tissue vs. liver) remain poorly defined. Genetic and pharmacological tools are needed to dissect their individual contributions to systemic glucose homeostasis. Emerging evidence indicates that RalA predominantly regulates insulin-stimulated glucose uptake and vesicular trafficking, particularly in adipose tissue, where it controls GLUT4 translocation and systemic glucose homeostasis [76].

#### 6.5.2. Investigation of Crosstalk with Other Metabolic GTPases

The Ras–Ral axis does not operate in isolation. Its integration with signaling from other small GTPase families—such as Rho proteins in cytoskeletal reorganization, Rabs in vesicle trafficking, and ARF proteins in lipid metabolism—warrants detailed explorations to understand the broader signaling network [77]. Additionally, ARF GTPases have been implicated in lipid metabolism and membrane curvature, linking Ral-mediated exocyst function to lipid droplet dynamics and hepatic metabolism [78].

#### 6.5.3. Examination of Roles in Diabetic Complications

Finally, future research should investigate whether pharmacological rebalancing of the Ras–Ral axis can ameliorate the microvascular and macrovascular complications of diabetes, such as nephropathy, retinopathy, and cardiovascular disease, which are driven in part by the same inflammatory and metabolic stresses that dysregulate this pathway. Addressing these multifaceted challenges will not only provide a rigorous test of the proposed hypothesis but also establish a concrete roadmap for developing a novel class of diabetes therapeutics aimed at correcting the underlying molecular pathophysiology.

## 7. Conclusions

DM, particularly Type 2 DM (T2DM), is characterized by a complex interplay of insulin resistance and β-cell dysfunction. This review has synthesized evidence positioning the Ras–Ral signaling axis as a central, yet dichotomous, regulator of these pathological processes. We propose a unifying hypothesis that T2DM progression involves a pathological imbalance within this axis, marked by hyperactive Ras-driven inflammatory and proliferative signals alongside insufficient Ral-mediated vesicular trafficking and secretory functions. This imbalance directly impairs GLUT4 translocation and insulin exocytosis, fueling hyperglycemia and metabolic decline.

The therapeutic implication of this model is a dual-pathway strategy that selectively inhibits pathogenic Ras signaling while concurrently enhancing beneficial Ral activity. Advances in direct Ras inhibition—including allele-specific inhibitors, farnesyltransferase inhibitors, and natural compounds—provide a growing arsenal for the first arm of this approach. For the second arm, enhancing Ral function via GEF activators, exocyst stabilizers, or RalGAP inhibitors presents a novel and promising, though underdeveloped, pharmacological frontier.

However, translating this conceptual framework into clinical reality presents significant challenges. These include mitigating potential oncogenic risk, achieving tissue and pathway selectivity, identifying responsive patient populations through biomarker discovery, and rigorously evaluating combination therapies in relevant disease models. Addressing these translational hurdles, as outlined, is essential for moving from mechanistic insight to viable therapy.

In summary, the Ras–Ral axis offers a mechanistically grounded, innovative strategy for diabetes treatment that moves beyond symptomatic glucose lowering to target core molecular drivers of disease. Future research focused on overcoming the outlined challenges will determine whether this axis can be harnessed to restore glucose homeostasis and β-cell health, paving the way for a new era of precision medicine in diabetes management.

## Figures and Tables

**Table 1:** Comparative Roles of Ras and Ral GTPases in Glucose Metabolism and Insulin Signaling.

Feature	Ras GTPases	Ral GTPases (RalA/RalB)	Delivery System Compatibility
Primary Molecular Function	Molecular switch for growth, survival, and proliferation signals [9].	Molecular switch for vesicle trafficking, exocytosis, and cytoskeletal dynamics [19].	Nanoparticle and lipid carrier-based delivery system [36]
Role in Diabetes Pathogenesis	Promotes insulin resistance via inflammation (NF-κB) and impaired insulin signaling (PI3K/AKT) [37,38].	Supports insulin secretion and GLUT4 translocation; deficiency or imbalance contributes to secretory dysfunction [39].	Tissue-targeted delivery system [40]
Key Downstream Pathways in Metabolism	MAPK/ERK, PI3K/AKT [11].	PI3K/AKT (indirect), Exocyst complex, RalBP1 [19]	Controlled-release polymeric delivery system [26]
Effect on GLUT4 Trafficking	Chronic activation inhibits translocation [41].	Activation (RalA) facilitates vesicle docking and fusion via the exocyst [42].	Stimuli-responsive hydrogels [27]
Effect on Pancreatic β-Cell Function	Hyperactivation can impair insulin synthesis and promote apoptosis [14].	Essential for insulin granule exocytosis; protective [39].	Glucose-responsive delivery platforms [43]
Inflammatory Modulation	Potent activator of pro-inflammatory pathways (e.g., NF-κB) [38].	Can suppress pro-inflammatory signaling; context-dependent [21].	Long-circulating nanocarriers [40]
Therapeutic Targetability	Inhibitors: FTIs (e.g., F-FTS), G12C allele-specific inhibitors (Sotorasib), natural compounds (Paeoniflorin) [14,37,44].	Enhancers: Underdeveloped; potential targets include RalGEF agonists, exocyst stabilizers, RalGAP inhibitors [39].	Co-delivery polymeric nanoparticles [36]

**Table 2:** Key Experimental Evidence Linking Ras–Ral Axis to Metabolic Phenotypes.

Experimental Model/Context	Key Intervention/Observation	Metabolic Outcome	Implication for Diabetes	Reference
In Vitro/Cellular Studies				
Pancreatic β-cells (INS-1, MIN6)	High glucose exposure → increased HRAS expression and apoptosis. Paeoniflorin treatment rescues this effect.	HRAS upregulation correlates with β-cell apoptosis; inhibition is protective.	Ras hyperactivation contributes to β-cell failure in glucotoxicity.	[14]
Adipocytes/Muscle cells	Insulin stimulation → RalA activation and exocyst recruitment to GLUT4 vesicles.	RalA is necessary for insulin-stimulated GLUT4 translocation.	RalA is a direct molecular link between insulin signaling and glucose uptake.	[42]
Animal Models (Rodent)				
High-Fat Diet (HFD) Mice	Administration of Ras inhibitor F-FTS.	Improved glucose tolerance, reduced serum insulin, attenuated adipose tissue inflammation.	Pharmacological Ras inhibition reverses key features of diet-induced insulin resistance.	[37]
Genetic Model (Mice)	β-cell-specific expression of active Ras (e.g., KRAS G12D).	Impaired glucose-stimulated insulin secretion, hyperglycemia.	Constitutive Ras activation in β-cells is sufficient to cause secretory dysfunction and diabetes.	[5]
Genetic Model (Mice)	Whole-body or adipose-specific RalGAPβ knockout.	Enhanced RalA activity, improved systemic glucose tolerance, increased insulin sensitivity.	Endogenous suppression of Ral activity (via RalGAPs) is a physiological regulator of insulin sensitivity.	[39]
Obesity Model (Mice)	Investigation of white adipocytes from obese mice.	Elevated inactive RalA (iRalA) promotes Drp1-mediated mitochondrial fission, leading to insulin resistance.	Obesity corrupts RalA signaling, switching its role from metabolic regulator to a promoter of mitochondrial dysfunction.	[46]
Human Association Studies				
Placental tissue from obese pregnancies	Elevated Ras activity correlates with inflammatory markers and altered nutrient transporters.	Links maternal obesity to fetal metabolic programming and insulin resistance.	Suggests Ras inhibition could benefit gestational metabolic disorders and break the cycle of intergenerational diabetes risk.	[50]

**Table 3:** Potential Therapeutic Agents Targeting the Ras–Ral Axis for Diabetes.

Agent Class/Name	Primary Target/Mechanism	Proposed Role in Diabetes	Key Supporting Preclinical Evidence (Reference)	Development Stage/Notes
Ras Inhibitors				
5-Fluoro-farnesylthiosalicylic acid (F-FTS)	Farnesyltransferase inhibitor; displaces Ras from membranes.	Improves insulin sensitivity, reduces inflammation.	Reduced serum insulin/glucose, suppressed NF-κB in HFD mice [37]	Preclinical (metabolic models).
Paeoniflorin	Natural monoterpenoid glycoside; downregulates HRAS expression.	Protects β-cells from glucotoxicity-induced apoptosis.	Dose-dependent HRAS downregulation and anti-apoptotic effect in β-cells [14]	Preclinical (in vitro/animal studies).
Sulindac sulfide	NSAID; inhibits Ras–Raf binding and GEF-mediated nucleotide exchange.	Potential to inhibit Ras-driven inflammation and proliferation.	Inhibited Ras-induced transformation in fibroblasts [58]	Limited by off-target effects; serves as a proof-of-concept scaffold.
Ral Enhancers (Proposed)				
RalGEF Agonists	Small molecules activating RalGDS or other Ral-specific GEFs.	Boost Ral-GTP levels, enhance insulin secretion and GLUT4 trafficking.	Genetic activation of Ral signaling improves glucose tolerance [39].	Hypothetical/early discovery stage; high-throughput screens needed.
Exocyst Stabilizers	Compounds stabilizing RalA–exocyst (Sec5/Exo84) interaction.	Directly facilitates insulin granule and GLUT4 vesicle docking/fusion.	RalA–exocyst interaction is essential for insulin-stimulated GLUT4 translocation [42].	Hypothetical; requires structural biology for rational design.
RalGAP Inhibitors	Inhibitors of Ral GTPase-Activating Proteins (RalGAPα/β).	Increase endogenous Ral-GTP lifetime and signaling.	RalGAPβ knockout mice exhibit enhanced RalA activity and improved insulin sensitivity [39].	Hypothetical; no specific inhibitors reported.
Dual-Action/Repurposed Agents				
Glycyrrhetinic Acid (GA)	Natural triterpenoid; modulates Ras/MAPK and PI3K/AKT balance.	Improves insulin signaling, reduces IRS-1 serine phosphorylation.	Enhanced insulin response in cellular models [64]	Preclinical; demonstrates principle of pathway rebalancing.

## Data Availability

No new data were created or analyzed in this study. Data sharing is not applicable to this article.
